# An excellent result of surgical treatment in patients with severe pulmonary arterial hypertension following mitral valve disease

**DOI:** 10.1186/s13019-015-0274-1

**Published:** 2015-05-13

**Authors:** Xiaochun Song, Cui Zhang, Xin Chen, Yongming Chen, Qiankun Shi, Yongsheng Niu, Jilai Xiao, Xinwei Mu

**Affiliations:** 1 Department of Intensive Care Unit (ICU), Nanjing Hospital Affiliated to Nanjing Medical University, Qinhuai district, No. 68 Changle Road, Nanjing, 210006 Jiangshu China; 2 Department of Thoracic and Cardiovascular Surgery, Nanjing Hospital Affiliated to Nanjing Medical University, Qinhuai district, No. 68 Changle Road, Nanjing, 210006 Jiangshu China

**Keywords:** Surgical treatment, Severe pulmonary arterial hypertension, Mitral valve disease

## Abstract

**Objective:**

Observe the efficacy of surgical treatment in patients with severe pulmonary arterial hypertension caused by mitral valve disease.

**Methods:**

We examined the results of surgical treatment in 32 patients with mitral valve disease and severe pulmonary arterial hypertension (pulmonary arterial systolic pressure ≥ 80 mmHg) retrospectively. Operative and postoperative data collection included type of the surgery, cardiopulmonary bypass time, cross-clamp time and the mortality rate. Pulmonary arterial systolic pressure, left atrial diameter, left ventricular end-diastolic diameter, and left ventricular ejection fraction were recorded and compared.

**Results:**

A total number of 32 patients had the operation of mitral valve replacement. Among those subjects, twenty-seven patients were surgically replaced with mechanical prosthesis and five patients with tissue prosthesis. Only one patient died of pneumonia, with a mortality rate of 3.1 %. The statistical results of preoperative and postoperative echocardiographic data showed significant decrease in pulmonary arterial systolic pressure (101.2 ± 20.3 versus 48.1 ± 14.3 mmHg, *P* < 0.05), left atrial diameter(67.6 ± 15.7 versus 54.4 ± 11.4 mm, *P* < 0.05) and left ventricular end-diastolic diameter (52.3 ± 9.5 versus 49.2 ± 5.9 mm, *P* < 0.05). There was no significant change in left ventricular ejection fraction (59.2 ± 6.5 versus 57.9 ± 7.6, *P* = NS). At the time of follow-up, twenty-eight (96.6 %) patients were classified in New York Heart Association functional class I or II, one(3.4 %) in class III, with the mortality rate is zero percent.

**Conclusions:**

Mitral valve replacement can be performed successfully in patients with mitral valve disease and severe pulmonary arterial hypertension with an acceptable perioperative risk.

## Background

Pulmonary arterial hypertension(PAH) increases the risk of complications in patients undergoing mitral valve surgery perioperatively, with a high operative mortality rate ranging from 15 % to 31 % [1, 2]. Many cardiac surgeons refused to operate on these high-risk patients. With the enhancement in medical technology and the development of pharmacological treatment, the operative risk in patients undergoing cardiac surgery with severe PAH should be decreased. Thus, in this study, we retrospectively examined patients with mitral valve disease and severe PAH treated surgically in our hospital over a 3-year period to assess the perioperative mortality rate, 3-year survival rate and survivors’ cardiac function through New York Heart Association (NYHA) functional class.

## Methods

### Study population

This prospective study was conducted between January, 2010 and December, 2012. Patients were identified through reviewing of operative reports and medical records. A total of 617 patients underwent mitral valve surgery in Nanjing First Hospital Affiliated to Nanjing Medical University during this period. Thirty-two patients who met the inclusion criteria with severe PAH following mitral valve disease in whom pulmonary arterial systolic pressure(PASP) were ≥ 80 mmHg on echocardiographic determination were enrolled in this study. Patients with chronic obstructive pulmonary disease(COPD) and coronary artery disease were excluded from the study. Informed consent was obtained from the patients before the study. The implementation of this study was authorized by the Hospital’s Ethics Committee.

### Data collection and definition

In this study, all patients were diagnosed with rheumatic heart disease, specifically, 24(75.0 %) patients with mitral stenosis, 5(15.6 %) with mitral regurgitation, and 3(9.4 %) with mixed lesions, 9(28.1 %) with critical aortic valve disease and 28(87.5 %) with moderate-severe tricuspid regurgitation. Severe mitral stenosis is defined as a mitral valve orifice area < 1.0 cm^2^. Severe mitral regurgitation is defined as a ratio of jet area to left atrial area > 40 %, and a mixed lesion met the criteria for both mitral stenosis and mitral regurgitation. Right ventricular hypertrophy is diagnosed on the basis of electrocardiography and 2-dimensional transthoracic echocardiography. General anesthesia and standard cardiopulmonary bypass are used for the operations in this study. Sevoflurane, propofol, cisatracurium besylate and remifentanil were adminisered during the period of anesthesia. All procedures were performed through a median sternotomy. Mild-moderate general hypothermia (28 °C–35 °C) was induced on these patients. All operations used cold antegrade oxygenated blood cardioplegia. The mitral valve was approached through the right atrium and interatrial septum in all patients. Posterior mitral leaflet was preserved as much as possible in 29(90.6 %) patients. Most of the patients were given milrinone and vasodilator drugs including nitroglycerine, prostaglandin E1,and fasudil until the hemodynamics stabilized. The use of inotropic agents (dopamine, dobutamine, and adrenaline) was depended on the patients’ hemodynamic status. Preoperative and postoperative transthoracic echocardiography was performed in all patients with the absence of pulmonary stenosis. Pulmonary arterial systolic pressure(PASP), left atrial diameter(LAD), left ventricular end-diastolic diameter(LVDd), and left ventricular ejection fraction(LVEF) were recorded and compared. Operative and postoperative data record included type of the surgery, CPB time, cross-clamp time, the duration of ventilatory support and the length of ICU stay, which were shown in Table 1.

**Table 1:** Results of surgery in 32 patients

Variable	Number	%
MVR(n)	1	3.1
MVR and TVP(n)	22	68.8
MVR and AVR(n)	3	9.4
MVR,AVR and TVP(n)	6	18.7
CPB time(minutes) [range]	119.9 ± 37.4[72–213]	
Cross-clamp time(minutes) [range]	82.5 ± 31.8[45–156]	
Duration of ventilatory support (hours) [range]	21.0 ± 31.5[3.8-131.0]	
NPPV (n)	6	18.8
Reintubation(n)	1	3.1
Reoperation(n)	2	6.3
Length of ICU Stay (days) [range]	2.4 ± 3.2[0.6-13.6]	

*MVR*, mitral valve replacement; *TVP*, tricuspid valvuloplasty; *AVR*, aortic valve replacement; *CPB*, cardiopulmonary bypass; *ICU*, intensive care unit; *NPPV*, noninvasive positive pressure ventilation

Follow-up was conducted by phone interview. Patients were questioned regarding their levels of activity, subsequent hospitalization, the occurrence of stroke, and the need for repeating cardiac surgery. In case of death occurred, family members were questioned as to the date and cause of death.

### Data analysis

Continuous variables were reported as mean ± standard deviation which were analyzed using the computer programmes SPSS for Windows, Version 11.5. The data of two groups were computed with a paired Student’s *t* test. Results were considered statistically significant if *P* value < 0.05.

## Results

### Perioperative mortality

This retrospective analysis of surgically treated patients with severe PAH and mitral valve disease demonstrated a perioperative mortality of 3.1 % and a survival rate of 96.9 % with a mean follow-up period of 26.3 ± 10.7 months (range, 12–45 months). Survivors demonstrated marked symptomatic improvement, with 96.6 % in NYHA functional classIor II.

### Baseline characteristics of the study population

The study population consisted of 32 patients, 71.8 % women, with a mean age of 53.1 ± 9.7 years (range, 37 to 71 years). Atrial fibrillation was presented in 27(84.4 %) patients. One patient underwent closed mitral valvuloplasty for mitral stenosis prior to the surgery. All patients were symptomatic, and the majority (90.6 %) were classified in NYHA functional class III or IV, 13(40.6 %) patients had electrocardiographic and echocardiographic evidence of right ventricular hypertrophy, and 21(65.6 %) patients appeared edematous under lower extremities. Table 2 summarized baseline characteristics of the study population.

**Table 2:** Baseline characteristics of 32 patients

Variable	Number	%
Age (years)[range]	53.1 ± 9.7[37–71]	
Male sex(n)	9	28.1
Female sex(n)	23	71.9
NYHA functional class		
II(n)	3	9.4
III(n)	23	71.9
IV(n)	6	18.7
Atrial fibrillation(n)	27	84.4
Prior closed mitral valvuloplasty(n)	1	3.1
Right ventricular hypertrophy(n)	13	40.6
Edema of lower extremities(n)	21	65.6
Mitral stenosis(n)	24	75.0
Mitral regurgitation(n)	5	15.6
Mitral stenosis and mitral regurgitation(n)	3	9.4
Severe tricuspid regurgitation(n)	28	87.5
Critical aortic valve desease(n)	9	28.1

*NYHA*, New York Heart Association

### Preoperative and postoperative echocardiographic parameters and postoperative swan-Ganz catheter parameters

The mean and standard deviation of postoperative pulmonary arterial systolic pressure were 44.4 ± 14.8 mmHg which derived from the Swan-Ganz catheter during intensive care unit. The statistical results of preoperative and postoperative echocardiographic data showed significant decrease in PASP(101.2 ± 20.3 versus 48.1 ± 14.3 mmHg, *P* < 0.05), LAD(67.6 ± 15.7 versus 54.4 ± 11.4 mm, *P* < 0.05) and LVDd(52.3 ± 9.5 versus 49.2 ± 5.9 mm, *P* < 0.05). There was no significant change in LVEF(59.2 ± 6.5 versus 57.9 ± 7.6, *P* = NS). Echocardiographic data were summarized in Table 3.

**Table 3:** Preoperative and postoperative echocardiographic parameters of 32 patients

Variable	Preoperative	Postoperative	*P* value
PASP(mmHg)	101.2 ± 20.3[81–159]	48.1 ± 14.3[26–77]	0.000
LAD(mm)	67.6 ± 15.7[48–105]	54.4 ± 11.4[39–80]	0.000
LVDd(mm)	52.3 ± 9.5[39–77]	49.2 ± 5.9[40–66]	0.042
LVEF(%)	59.2 ± 6.5[42–68]	57.9 ± 7.6[31–70]	0.375

*PASP*, pulmonary arterial systolic pressure; *LAD*, left atrial diameter; *LVDd*, left ventricular end-diastolic diameter; *LVEF*, left ventricular ejection fraction

### Results of surgery

Thirty-two patents were operated for mitral valve replacement (MVR) with 27 cases of mechanical prosthesis and 5 cases of tissue prosthesis. Additional surgical procedures included aortic valve replacement(AVR) which was performed in 9(28.1 %) patients,and tricuspid valvuloplasty(TVP) which was performed in 28(87.5 %) patients with severe tricuspid regurgitation, 2 cases of Devega annuloplasty, 10 cases of Key annuloplasty and 16 cases of Balmedic “C”ring placement. The CPB time was 119.9 ± 37.4 min (range, 72–213 min), the aortic cross-clamp time was 82.5 ± 31.8 min (range, 45–156 min),the duration of ventilatory support was 21.0 ± 31.5 h (range, 3.8–131 h), and the length of ICU stay was 2.4 ± 3.2 d (range, 0.6–13.6 d). One patient had bleeding complication requiring emergency reoperation after cardiac surgery. One patient received reoperation due to the pericardial effusion in the eighth day after cardiac surgery. A patient was intubated again after extubation of endotracheal tube for acute respiratory failure following left heart failure. Noninvasive positive pressure ventilation was applied to six patients with respiratory insufficiency in order to avoid reintubation in perioperative period.

### Survival analysis

Two patients were absent during follow-up. NYHA functional status was improved by one class or more in 29(93.5 %) patients after cardiac surgery during a mean follow-up period of 26.3 ± 10.7 months (range, 12–45 months). At the time of follow-up, twenty-eight patients (96.6 %) were in NYHA functional class I or II, one(3.4 %) was in class III, and none was in class IV. The mortality rate of the surgery is zero percent. Two patients were hospitalized for the second time due to stroke and right tibial fracture during the follow-up period.

## Discussion

PAH is a common complication of severe mitral valve disease [3, 4]. Raised pulmonary arterial pressure initially results from increased left atrial pressure, pulmonary arteriolar vasoconstriction, and ultimately obliterative changes in the pulmonary vascular bed. Excessive thickening of the media and intimal fibrosis of small muscular pulmonary arteries are typical changes of long-term mitral stenosis as well as other conditions associated with severe PAH [5]. In our study, the statistics of preoperative and postoperative echocardiographic data showed significant decline in PASP(101.2 ± 20.3 versus 48.1 ± 14.3 mmHg, *P* < 0.05), LAD(67.6 ± 15.7 versus 54.4 ± 11.4 mm, *P* < 0.05) and LVDd(52.3 ± 9.5 versus 49.2 ± 5.9 mm, *P* < 0.05) after mitral valve replacement which led to the complete resolution of mitral stenosis or regurgitation. The retraction of the left atrium and left ventricle decreases the pulmonary arterial pressure which further improves the function of the right ventricle.

PAH has been considered a risk factor in patients undergoing mitral valve replacement, with a high operative mortality rates. Najafi et al. [6] found the degree of PAH is strongly associated with perioperative mortality rate, ranging from 16 % in patients with mild PAH to 61 % in patients with systemic PAH. Several reports had demonstrated an improved outcome of the perioperative mortality rate with the times, from 5.6 % to 12 %, in patients with PAH underwent mitral valve replacement [7–11]. In our study, all patients with mitral valve disease and severe PAH were treated surgically in our hospital with a decreased perioperative mortality of 3.1 %,which was a satisfactory outcome. The improved outcome can be managed through the following respects. First of all, it is very important to improve preoperative cardiac function. All patients admitted to hospital were given cardiotonic, diuretic, vasodilator and oxygen therapy before cardiac surgery in this study. Secondly, selection of suitable type of mitral prosthetic valve is crucial to avoid acute left heart failure. The posterior mitral leaflet and mitral subvalvular apparatus were preserved as much as possible in mitral valve surgery since it is beneficial to maintain normal left ventricular geometry, stabilize and even improve left ventricular systolic and diastolic functions. Incidence of postoperative low cardiac output syndrome can be reduced as well. In this study, there was no significant difference in LVEF(59.2 ± 6.5 versus 57.9 ± 7.6, *P* = NS). Result of LVEF demonstrated left ventricular systolic function was not impaired by mitral valve replacement. Tricuspid annuloplasty was performed in 28(87.5 %) patients with severe tricuspid regurgitation. This procedure alleviates tricuspid regurgitation, decreases the preload of right ventricle and avoids the occurrence of the right ventricular dysfunction. Thirdly, comparing with left ventricle, right ventricular muscle contraction force is weaker, which means the self-regulation becomes poor when preload or afterload of right heart increase. Therefore, it is crucial to decrease cardiac preload and afterload of right heart through the usage of diuretics and vasodilators. Fluids management should be done under closed hemodynamic monitoring. Fluid restriction must be strictly followed once the hemodynamics became stable after cardiac surgery. Diuretic therapy is a good choice for reducing preload in both preoperative and postoperative period. More attention should be paid on to decreasing afterload of heart after mitral valve replacement in patients with severe PAH. If an inotropic agent is required, phosphodiesterase inhibitor is a better choice (e.g., milrinone) to increase cAMP concentration and improve myocardial contractility and diastolic relaxation. Furthermore, the vasodilator effect of milrinone can reduce afterload and pulmonary arterial pressure [12]. Fourthly, pulmonary arterial pressure decrease significantly after mitral valve replacement with relief of mitral stenosis or regurgitation. However, many factors including pain, anxiety and anoxia are still present which can increase pulmonary arterial pressure after cardiac surgery. Therefore, moderate sedation and analgesia are beneficial to patients. Lastly, postoperative mechanical ventilation is the first step to avoid occurrence of hypoxia. When the tissue becomes hypoxic, pulmonary arteriole starts to constrict and pulmonary arterial pressure increases. Ventilator associated pneumonia(VAP) is one of the most serious but common complication of patients with mechanical ventilator. This will not only prolong the hospital stay but also burden the medical expenses of the patient. The use of noninvasive positive pressure ventilation(NPPV) can shorten the duration of ventilatory support, decrease the incidence of ventilator associated pneumonia and reduce reintubation rates. Furthermore, the application of noninvasive positive pressure ventilation in the patients with respiratory insufficiency can reduce pulmonary interstitial edema, improve lung compliance and supply enough oxygen. In this study, six patients were treated with noninvasive positive pressure ventilation and none of those experienced further need for invasive mechanical ventilation.

Other risk factors for patients undergoing mitral valve surgery include age [10, 13, 14] and left ventricular ejection fraction [15, 16]. In this study, the mean age were 53.1 ± 9.7 years and mean LVEF were 59.2 ± 6.5 %, which contributed to the decreased perioperative mortality of 3.1 %.

Mitral valve repair offers several advantages which include the avoidance of long-term anticoagulation and the preservation of the continuity between the mitral annulus and papillary muscles. Mitral valve repair should be Superior to mitral valve replacement for mitral regurgitation [17]. However, it was rarely performed because of the high rate of repair failure in patients with rheumatic etiologic mitral regurgitation as well as severe subvalvular pathology with heavy calcification of leaflets [18, 19].

NYHA functional status was improved by one class or more in 29(93.5 %) patients and no incidence of death occurred during a mean follow-up period of 26.3 ± 10.7 months (range, 12–45 months).

### Limitations of the study

The limitations of our study are the small sample size and short follow-up interval. The small number of subjects limits the statistical power of the study. With regard to the short follow-up period, it cannot demonstrate long-term survival rate and living quality of the patients. Despite these limitations, this study results showed an improved outcome of surgical treatment in patients with severe pulmonary arterial hypertension following mitral valve disease. Surgical management should be encouraged to more cardiac surgeons to appreciate its better effect on those high-risk patients.

## Conclusions

This study showed that mitral valve replacement can be performed successfully in patients with mitral valve disease and severe pulmonary arterial hypertension with an acceptable perioperative risk. In terms of long-term surgical outcome, majority of survivors experienced symptomatic improvement through surgery.

## Abbreviations

ICU: Intensive care unit
NYHA: New York Heart Association
CPB: Cardiopulmonary bypass
COPD: Chronic obstructive pulmonary disease
PAH: Pulmonary arterial pressure
PASP: Pulmonary arterial systolic pressure
LAD: Left atrial diameter
LVDd: Left ventricular end-diastolic diameter
LVEF: Left ventricular ejection fraction
MVR: Mitral valve replacement
AVR: Aortic valve replacement
TVP: Tricuspid valvuloplasty
VAP: Ventilator associated pneumonia
NPPV: Noninvasive positive pressure ventilation
